# TIM-3 Regulates Distinct Functions in Macrophages

**DOI:** 10.3389/fimmu.2016.00229

**Published:** 2016-06-13

**Authors:** Ranferi Ocaña-Guzman, Luis Torre-Bouscoulet, Isabel Sada-Ovalle

**Affiliations:** ^1^Laboratorio de Inmunología Integrativa, Instituto Nacional de Enfermedades Respiratorias “Ismael Cosío Villegas”, México City, México; ^2^Departamento de Fisiología Respiratoria, Instituto Nacional de Enfermedades Respiratorias “Ismael Cosío Villegas”, México City, México

**Keywords:** TIM-3, macrophages, immune regulation, innate immune response, tolerance mechanisms

## Abstract

The transmembrane protein TIM-3 is a type I protein expressed by sub-types of lymphoid cells, such as lymphocytes Th1, Th17, Tc1, NK, as well as in myeloid cells. Scientific evidence indicates that this molecule acts as a negative regulator of T lymphocyte activation and that its expression is modified in viral infections or autoimmune diseases. In addition to evidence from lymphoid cells, the function of TIM-3 has been investigated in myeloid cells, such as monocytes, macrophages, and dendritic cells (DC), where studies have demonstrated that it can regulate cytokine production, cell activation, and the capture of apoptotic bodies. Despite these advances, the function of TIM-3 in myeloid cells and the molecular mechanisms that this protein regulates are not yet fully understood. This review examines the most recent evidence concerning the function of TIM-3 when expressed in myeloid cells, primarily macrophages, and the potential impact of that function on the field of basic immunology.

## Introduction

The proteins of the TIM family (T cell immunoglobulin and mucin domain) are expressed on the surface of diverse cell types of the immune system (1). They were first described in mice T lymphocytes (TIM-1, TIM-2, TIM-3, and TIM-4) and next in humans (TIM-1, TIM-3, and TIM-4) (2–5). Homologs of TIM proteins have been identified in other species as well, including monkeys and rats (2, 6). These proteins can regulate diverse effectors pathways of the immune response. TIM-3 is expressed in CD4+ T cells that secrete interferon-gamma (IFN-γ), CD8+ T cells, dendritic cell (DC), and macrophages, among others. Also, it has been proposed as a regulator of the Th1 type immune response (7). Evidence gathered to date suggests both negative (inhibition) and positive (activation) regulatory functions on diverse components of the immune response. These data underline the importance of determining whether the function of TIM-3 differs in the diverse cell types in which it is expressed.

The first protein of this family discovered (TIM-1) was described as a marker of renal damage and associated with adhesion functions in a murine model (3, 4). The association of TIM proteins with immunological functions began to be deduced from a study that compared the presence of polymorphisms in BALB/C and DBA/2 mice by the genetic mapping of congenic animals, in order to identify the locus responsible for variations in susceptibility to asthma (5). That study confirmed the presence of the locus called T cell and airway phenotype regulator (Tapr) that groups a series of genes found in a region previously associated with susceptibility to asthma (5), which includes, among others, genes of the TIM family (5). Since then, the function of these proteins has been studied in several *in vivo* and *in vitro* experimental models to evaluate its participation during the immune response of autoimmune diseases, bacterial and viral infections, and pathologies with oncogenic origin.

## Structure of the TIM Proteins

In mice, the family of TIM proteins includes eight genes, of which only four codify for functional proteins (TIM-1 to TIM-4). In humans, three genes have been identified that codify for TIM-1, TIM-3, and TIM-4 (8). Each one of these genes codifies for a type I protein localized in the cell’s plasma membrane. The TIM-3 protein is 281 amino acids in size, with a total homology of 77% with the murine protein (7). The structure of the TIM proteins consists of four well-defined regions.

1. Variable immunoglobulin domain (IgV),
2. Mucin domain,
3. Transmembrane region, and
4. Intracellular stem.

**Figure 1:**
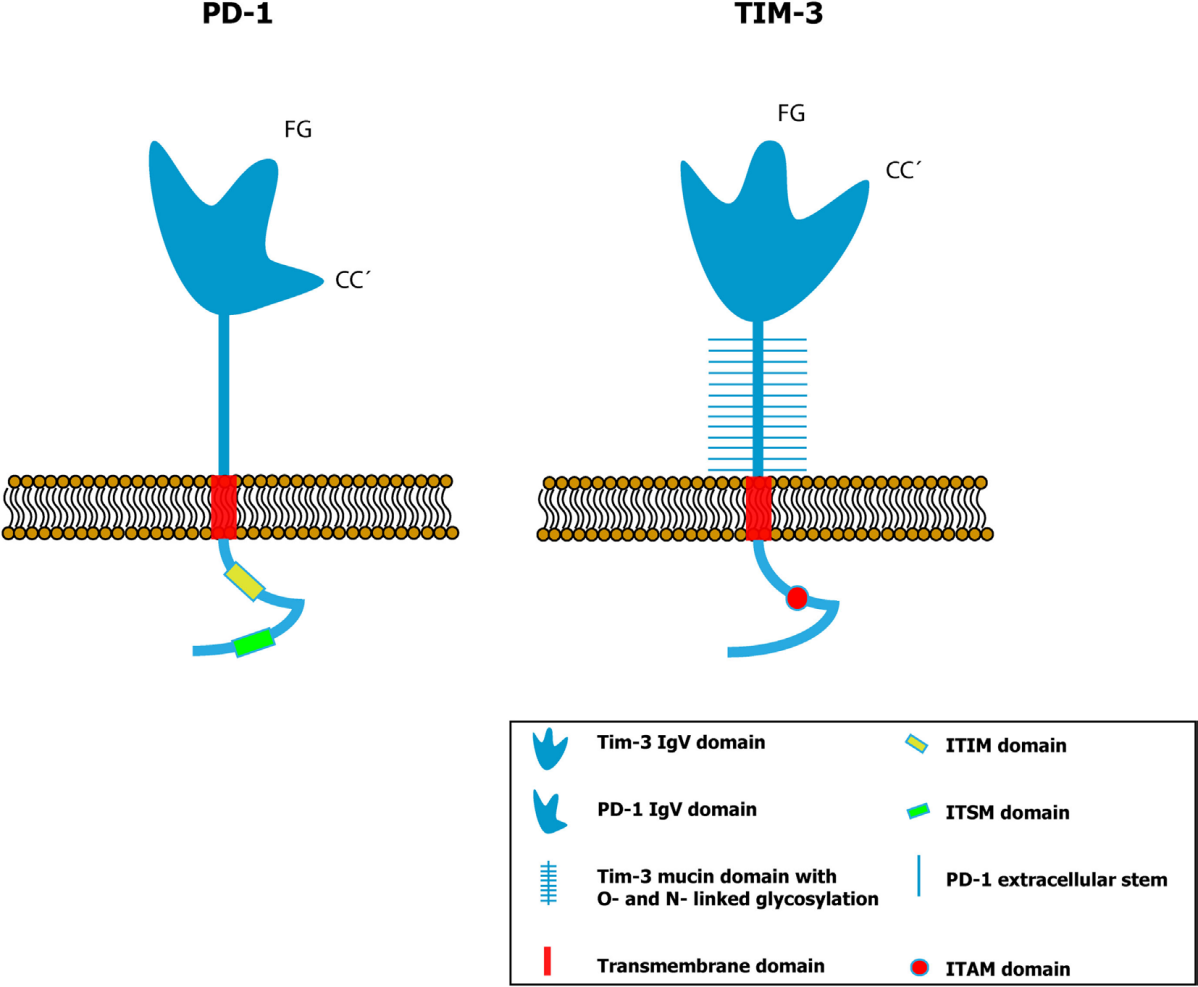
**Comparative schemes of the PD-1 (left panel) and TIM-3 (right panel) proteins showing the different conformation of the variable immunoglobulin domain (IgV)**. In a conventional IgV domain like that of PD-1, the FG and CC′ loops are spatially distant (25 Å), while in the IgV domain of TIM-3, the distance between them is smaller due to the presence of disulfide bonds. Thus, a distinct spatial conformation is formed in TIM-3 compared to the other proteins that contain immunoglobulin domains (9, 17). [Glycosylation sites within the IgV domain (9) have been omitted to simplify.]

The IgV domain contains two anti-parallel chains, where four cysteines can be identified in the variable region of all members of this family, suggesting that they are highly conserved regions. Although it shares some characteristics with the IgV domains of a more conventional structure, the IgV domain of TIM-3 contains two disulfide bridges in the four cysteines conserved from the TIM proteins (9), a characteristic shared by the entire TIM family. In most IgV domains of diverse proteins, there are well-defined structures called CC′ loop and FG loop, named after the folded beta sheets that make up interactions established by the disulfide bonds. In the super-family of conventional immunoglobulins, the CC′ and FG loops are located at opposite ends of the IgV domain, at an approximate distance of 25 Å, but in TIM-1, 3, and 4 proteins, a distinct spatial arrangement has been identified due to the presence of disulfide bonds formed between the four cysteines, which re-orient the CC′ loop more proximal to the FG loop, creating a unique cleft in the IgV domain of the proteins of this family (9) (Figure 1). This characteristic cleft of the TIM proteins is stabilized by disulfide and hydrogen bonds (9). It is possible that this peculiar structure may modify the biological function of the IgV domain of TIM-3, though this has not yet been demonstrated. The two ligands known for TIM-3 – galectin-9 (Gal-9) and phosphatidylserine (PS) – bond to the protein’s IgV domain. Although there are glycosylation sites at the extracellular domain of TIM-3, it has been shown that only IgV glycosylation is required for Gal-9 binding (10). On the other hand, the PS binding to TIM-3 does not require additional glycosylations (9, 11).

The mucin domain varies considerably in length among different members of this family. TIM-3 is the molecule with the smallest domain (8). This region is especially rich in threonine, proline, and serine (9). TIM proteins cross the cell membrane by means of a tail embedded in the lipid bilayer. This tail is formed primarily of hydrophobic amino acids and penetrates into the interior of the cell with a cytoplasmic domain of ~42–77 amino acids in size. This is the most similar domain in humans and mice (8). Within the intracellular region of TIM-1 and TIM-3, there are phosphorylation sites, but in the former case, they are of two sub-types: one called TIM-1a that is expressed mainly in human liver cells and does not contain the amino acid sequence on which the kinase enzyme acts and the other, TIM-1b, is found primarily in human kidneys and conserves two threonine residues that make it susceptible to phosphorylation (8).

Based on the knowledge that TIM-2 transduces intracellular signals by phosphorylation of tyrosine residues, it was considered that this mechanism could also be present in TIM-3 (12). It has been demonstrated that the intracellular signaling of TIM-3 begins after its interaction with Gal-9 and when the tyrosine 265 (Y265) located at the cytoplasmic tail is phosphorylated by the interleukin inducible T-cell kinase (ITK) (13). Additionally, it has also been documented that the kinases Fyn and Lck can mediate a more efficient phosphorylation of TIM-3 (13). Finally, Rangachari et al. demonstrated that the Bat-3 protein (human leukocyte antigen B-associated transcript 3) is associated with the intracellular portion of TIM-3 in T lymphocytes and Bat-3 recruits a kinase of the Src family (Lck) (14). Thus, determining whether the intracellular signaling of TIM-3 in the macrophage involves Bat-3 and some kinase of the Src family will be fundamental as we strive to achieve a better understanding of the functioning of this protein. There is evidence to suggest that the Lck protein is not expressed in macrophages, but may express some isoform; therefore, the functioning of the TIM-3 receptor could differ in cells of myeloid origin. Conducting research in this area will allow us to learn more about the differences that exist in the function of TIM-3 between lymphocytes and myeloid cells. All these evidences allow us to consider that intracellular phosphorylation of TIM-3 is mediated by different kinases and with different levels of efficiency. This final regulation may have a direct impact on TIM-3 function when expressed by different cells.

## TIM-3 and Its Expression in Macrophages

Although the TIM-3 protein was initially identified as a membrane marker specific for Th1 and Tc1 (7) lymphocytes, its expression was soon confirmed in other cells (Table 1) (7, 15, 16). Today, we know that it is expressed in monocytes, macrophages, DC, NK cells, and even in a variety of cells in different types of tumors (3, 13–15, 17). Scientific findings suggest that TIM-3 and its ligand Gal-9 function as a negative regulatory pathway in T cell activation. The failure, or absence, of this pathway has been associated with the development of autoimmune diseases (18–22); however, it is important to determine its function in other cells, such as monocytes or macrophages, since some reports suggest that TIM-3 can function as a molecule that participates in cell activation and the elimination of different pathogens (23, 24).

**Table 1:** **Functions of TIM-3 in the immune response**.

Cell type	Mechanism mediated or regulated by TIM-3	Reference
Macrophages–monocytes	Capture and elimination of apoptotic bodies after phosphatidylserine union	(29, 31)
	Inhibition of the TLR-mediated activation	(34, 49)
	Increases the production of IL-12, IL-6, and IL-10	(34, 35)
	High TIM-3 level of expression inhibits IL-12 production	
	Antigen cross-presentation	(29)
	Production of IL-1b dependent on caspase-1 activation. Elimination of intracellular M.tb	(23, 24)
Dendritic cells	Inhibits the immune response to damage signals as nucleic acids and HMGB-1 protein	(16)
	Capture and elimination of apoptotic bodies and antigen presentation	(29)
NK cells	Increased frequency of TIM-3+ cells induces immune tolerance and reduces inflammatory pathology in mice infected with *Toxoplasma gondii*	(48)
HeLa cells	Promotes metastasis	(37)

*CD, cluster of differentiation; DC, dendritic cell; HMGB1, high mobility group box 1 protein; IL, interleukin; MMP-9, matrix metalloproteinase 9; M.tb, *Mycobacterium tuberculosis*; NK, natural killer cell; TAM, tumor-associated macrophage; TIM-3, T cell molecule with immunoglobulin and mucin domain 3; TLR, toll-like receptor, VEGF, vascular endothelial growth factor*.

## TIM-3 Allows Recognition and Phagocytosis of Apoptotic Bodies via Phosphatidylserine

Efferocytosis or elimination of apoptotic cells and bodies is a physiologic and vital mechanism that avoids tissue inflammation and autoimmune responses. It was first described by de Cathelineau and Henson (25) to differentiate between macropinocytosis and opsonization. Efferocytosis is a mechanism that maintains immune tolerance while allowing development of antibacterial immune responses (26, 27). Recent evidence suggests that TIM proteins play a role in efferocytosis. The receptors utilized by phagocytic cells to internalize the detritus of apoptotic cells include some members of the TIM family. TIM-1 and 4 were the first proteins of this family in which the bonding site to PS was identified. PS is a phospholipid that translocates to the external face of the plasma membrane of apoptotic cells, and the principal “eat me” signal that triggers their capture and elimination (28). This early evidence suggested that TIM-3 could bond to phospholipids. Since then, crystallography studies have proven that TIM-3 does indeed bond to PS *via* an FG loop located in the IgV domain (9, 29). This mechanism was identified initially in CD8+ DC and macrophages in an *in vitro* model, and its presence was confirmed later in a murine model. Since PS is the principle signal for the phagocytosis of apoptotic bodies or cells, when recognition of this TIM-3-mediated phospholipid is blocked with specific antibodies apoptotic bodies are not removed, and this can induce immunological abnormalities, such as the generation of autoantibodies (29, 30). This scientific evidence agrees with the results obtained on lymphocytes upon evaluating the function of TIM-3 in autoimmune-type diseases, such as experimental autoimmune encephalitis (EAE) and multiple sclerosis, where research has documented that reducing TIM-3 expression is associated with an aggravated state of the disease and a high frequency of autoreactive cells (18). While these studies support the function of TIM-3 promoting immunological tolerance, they also raise new questions about this protein. For example, it is necessary to clarify why CD8+ DC can capture, phagocyte, and favor antigen presentation, while CD8− DC do not perform these functions although they also express TIM-3 and are capable of responding to the presence of apoptotic bodies by producing inflammatory cytokines (29). Studies have also confirmed that allelic variants of TIM-3, that differ in seven amino acids in the FG loop and come from two strains of mice (BALB/c and HBA), possess distinct abilities to interact with PS (31). It has been demonstrated that phagocytic cells from the BALB/c strain bond to, and phagocyte, apoptotic cells by means of TIM-3, while the cells that express the HBA variant of TIM-3 can form complexes with PS, but are incapable of internalizing apoptotic bodies (31). But these findings are not enough to clarify the differences in the function of TIM-3 in the different cell types that express it. For example, TIM-3 expression has been demonstrated on peritoneal exudative macrophages, but not on peritoneal resident macrophages. However, TIM-4 is highly expressed on peritoneal resident macrophages and binds to PS on apoptotic cells from the peritoneal cavity. The different expression profile and function of TIM-3 compared with TIM-4 is not redundant and is important for maintaining immune tolerance. These results may indicate that different macrophage subsets could use different molecules (TIM-3 or TIM-4) to recognize PS (29).

Available evidence seems to suggest that the function of TIM-3 changes depending on the type of cell in which it is expressed but that the level of expression seems to contribute as well; for example, CD8+ DC have a level of expression of TIM-3 three times greater than CD8− DC (29). Moreover, phagocytic activity of each cell subset is the result of various considerations as the level of expression of the phagocytic receptors, the microenvironment where the cells are located as well as their cytoskeletal architecture. For example, when TIM-3 is expressed by lymphocytes (32), these cells do not recognize apoptotic cells or bodies; however, macrophages or DCs expressing TIM-3 do phagocyte. For this reason, it is important to conduct functional studies comparing TIM-3+ vs. TIM-3− cells and identify the signaling pathways that define the role of this protein in each cell type.

## TIM-3 Regulates the Production and Release of Cytokines in Monocytes and Macrophages

**Figure 2:**
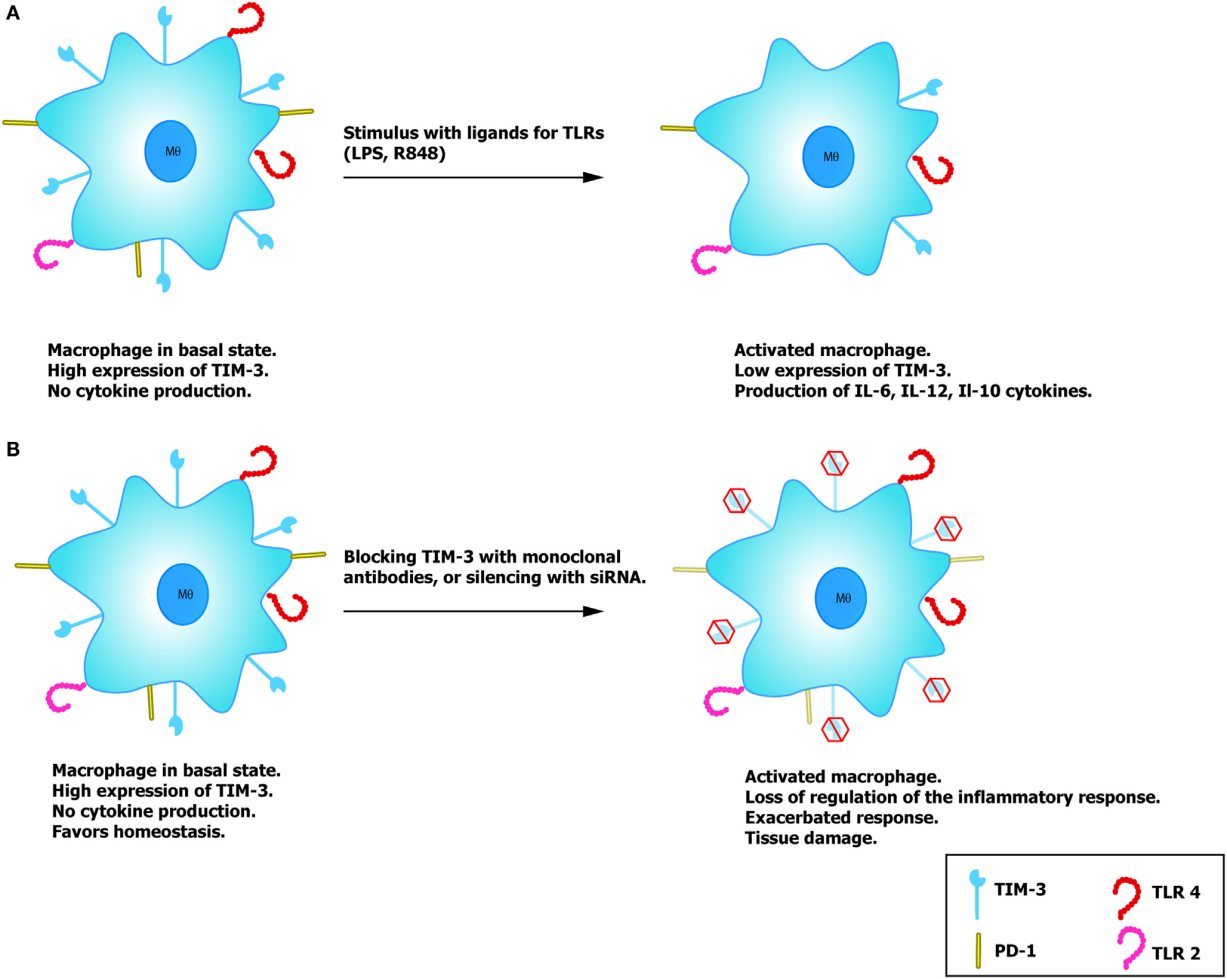
**Potential role of TIM-3 on macrophages**. **(A)** Macrophages with high expression of TIM-3 do not produce cytokines, favoring immune tolerance and reduce TLR activation. In the presence of the TLR ligands, LPS and R848, macrophage activation reduces expression of TIM-3 and lessens the regulatory effect mediated by this molecule, allowing correct activation and the consequent production of cytokines. **(B)** By silencing the expression of TIM-3 with siRNA or blocking antibodies, it is possible to induce correct macrophage activation similar to that observed on T cells.

It has been demonstrated that TIM-3 expression is dynamic, regulates the activation of diverse cells, and has the capacity to induce the production of distinct cytokines. This has been shown for monocytes and macrophages that express TIM-3 in a constant manner (Figure 2). In both the THP-1 cell line and CD14+ cells purified from human peripheral blood, studies have confirmed that monocytes/macrophages in a quiescent state have a high expression of TIM-3 with low cytokine production. When stimulated *in vitro* with LPS and the R848 compound (ligands for TLR4 and TLR7/8, respectively), these macrophages reduce the expression of TIM-3 in a concentration-dependent manner, while also beginning to produce IL-12 after stimulus reception (33). Blocking TIM-3 with monoclonal antibodies and posterior stimulation with TLR results in an increase in the production of IL-12, IL-6, and IL-10, but a reduced expression of PD-1, an inhibitor molecule of the T cell function. However, experiments made on THP-1 macrophages demonstrated that after silencing TIM-3 expression with siRNA, the production of IL-12 and IL-10 was increased (33, 34). These findings confirm that IL-12 expression is negatively regulated by TIM-3, contrary to what occurs with PD-1, which increases together with TIM-3. Another important finding is that when TIM-3 is expressed on other cells (*trans*) or on the macrophage itself (*cis*), the association of TIM-3 with its ligand, Gal-9, has a distinct effect as a regulator of TLR-mediated activation (35). For example, the *trans* association of TIM-3 and Gal-9 negatively regulates TLR signaling, leading to an IL-12 reduction, increase in the IL-23 production, decrease in the phosphorylation of STAT-1, and/or activation of STAT-3. Meanwhile, the association in *cis* favors the correct TLR signaling, and it has been demonstrated that expression of Gal-9 increases through this mechanism (35).

## TIM-3 in Macrophages and Cancer

Unlike the negative regulator PD-1, which has been proposed as a therapeutic target in different types of cancer, the function of TIM-3 in cancer has just begun to be studied, so we know very little about its role in this pathology. However, the fact that its function and changes in the profile of cellular expression in pathological states is similar to those of PD-1 suggests that its function in the tumor microenvironment should be initially evaluated in the same context. We know that TIM-3 expression is not restricted to mononuclear cells, because it has also been detected in cancer cells. TIM-3 can be expressed in distinct types of cancer cells, including myeloid leukemia, sarcoma, gastric, cervical and pulmonary cancer, and osteosarcoma, among others (36–39). In many of these types of cancer, the increased expression of TIM-3 has been associated with disease progression and shorter survival.

**Figure 3:**
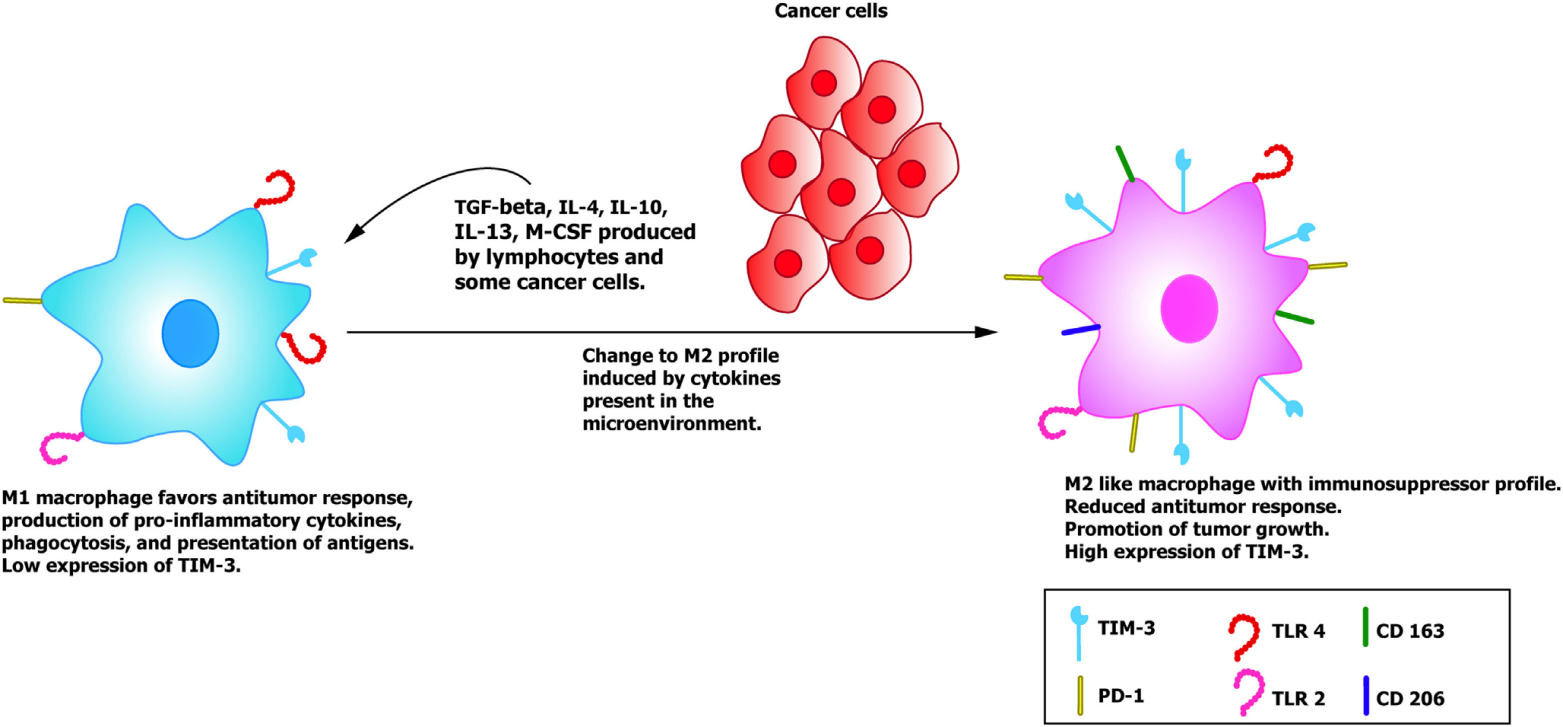
**TIM-3+ macrophages and its role in cancer progression**. The M1 macrophages recruited to eliminate cancer cells have a low expression of TIM-3 and perform diverse functions, such as phagocytosis, presentation of antigens, and production of pro-inflammatory cytokines, such as IFN-γ, TNF-α, and IL-12. When cancer cells and lymphocytes release anti-inflammatory cytokines into the environment such as IL-4 and TGF-β, they induce a change in the macrophages toward a regulator or tolerogenic profile (M2), accompanied by an increase in TIM-3 expression and in the expression of the specific markers of the M2 phenotype, such as DC 163 and DC 206, among others (41, 50, 51).

In T lymphocytes, it has been documented that the interaction of TIM-3 with Gal-9 inhibits cell activation and induces apoptosis *via* a mechanism that involves calpain and caspase-1 (40). Hence, the activity of the T lymphocytes could modify the tumor microenvironment, but how does TIM-3 participate in cancer progression? and what is its role in the tumor microenvironment when this molecule is expressed in macrophages and DC? These issues remain to be clarified. With respect to the innate immune system, studies have been conducted with distinct types of cancer to analyze the role of TIM-3 as a negative regulator of the function of T lymphocyte-mediated adaptive immunity and innate response when this molecule is expressed in macrophages and DC. One murine model of hepatic cancer documented an increase in TIM-3 expression in peripheral blood monocytes and tumor-associated macrophages (TAMs), a finding that was positively correlated with disease progression (41). TAMs are characterized by presenting a phenotype denominated M2, characterized by the expression of CD163, CD206, Arg-1, and IL-10 (Figure 3) (41). Scientific evidence suggests that this phenotype, together with the increase in the expression of TIM-3, are induced by the TGF-β (41) and other cytokines, such as IL-4, IL-10, IL-13, and M-CSF found in the tumor microenvironment [reviewed by Allavena et al. (42)], which favor the alternative activation of macrophages. It is feasible that the M1 type macrophages that are activated by IFN-γ and that perform innate functions as phagocytosis, promoting antitumor responses, and eliminating pathogens and transformed cells, could re-direct their M1 phenotype to the M2 phenotype, since they lose or reduce many of the protective functions in cancer, thus promoting tumor development and progression (41). These events are enabled by the characteristic plasticity of macrophages that – unlike lymphocytes which are polarized toward one of the known effector profile and suffer significant modifications at the level of the chromatin – show a genetic profile that changes depending on the type, concentration, and time of exposure to diverse stimuli (43, 44).

Other mechanisms that may favor tumor development involve exhausted T lymphocytes and DC. There is evidence that *in vitro* interaction between monocytes and macrophages with autologous T lymphocytes with senescent phenotype induces higher production of pro-inflammatory cytokines, such as TNF-α, IL-1b, and IL-6, as well as a greater production of angiogenic molecules, such as VEGF-α, MMP9, and IL-8, compared to monocytes cocultivated with control cells (45). TIM-3 can also favor the development of metastasis through a still unknown mechanism. For example, it has been reported that the HeLa cell line reduces the capacity for metastasis when TIM-3 is silenced by antisense cDNA (37).

In summary, TIM-3 participates in diverse suppressor pathways of the antitumor response in both myeloid cells and lymphocytes. As in the case of infectious diseases, it has been documented that simultaneous blocking of TIM-3 and PD-1 is more efficient in restoring the functionality of exhausted lymphocytes (46) and perhaps also in counteracting some inhibitory molecules in the macrophages and DC. It is possible that in macrophages, TIM-3 functions as a receptor that, upon being treated with antibodies or agonist molecules, induces a more efficient immunological response due to an increase of the inflammatory response, similar to that observed in T lymphocytes when treated with anti-PD-1 and anti-TIM-3 antibodies.

## TIM-3 Regulates the Immune Response in Infectious Diseases

The innate immune response to bacterial and viral infections is one of the most important mechanisms for controlling the pathogens that invade organisms every day. TIM-3 participates significantly in the response to these pathogens, since we know that it can regulate the activation of macrophages and the effectors function of these cells. Chronic bacterial infections like that of the intracellular pathogen *Mycobacterium tuberculosis* (M.tb) require a highly efficient Th1-type response; however, M.tb has developed diverse evasion strategies that allow it to survive the macrophage’s elimination systems, which are generally activated by IFN-γ produced by T lymphocytes with the Th1 profile. In an experimental model of *in vitro* infection with M.tb (H37Rv) performed in 2010, Jayaraman et al. demonstrated that when peritoneal macrophages were infected with M.tb (in the absence of T lymphocytes) and treated with TIM-3 in the form of a fusion protein, which is composed of the immunoglobulin V portion of mouse TIM-3 fused with the human IgG1 Fc tail to form the soluble TIM-3–Ig fusion protein, this protein limited bacterial growth through a mechanism that involved caspase-1-dependent IL-1b production (24). Blocking the activation of caspase-1, or treatment with IL-1b-blocking antibodies, eliminated the effect of the treatment with the TIM-3 fusion protein (24). This phenomenon has also been identified in human macrophages (23) infected with M.tb-H37Rv; moreover, studies have confirmed that IL-1b is the principal soluble factor that mediates inhibition of the growth of M.tb (47).

Another murine model, this one with infection by *Toxoplasma gondii*, an intracellular parasite that induces a Th1-type response, identified that mice of the C57BL/6 strain (susceptible) have a lower frequency of TIM-3+ immune cells in the spleen and mesenteric lymphatic nodes (NK, CD3+, CD11b, and CD11c+) compared to resistant BALB/c mice (48). The C57BL/6 mice developed an increased inflammatory condition mediated primarily by IFN-γ that induced extensive tissue damage in the small intestine with symptoms similar to Crohn’s disease (48). In contrast, the BALB/c mice that expressed a higher frequency of TIM-3+ cells did not develop this type of damage in the small intestine. These results suggest that TIM-3 functions as a molecule that favors tolerance by preventing an exacerbated inflammatory response in diverse tissues. One of the mechanisms by which TIM-3 can promote immunological tolerance in cells of the innate immune system consists in negatively regulating the activation induced by TLR signaling (35). This mechanism of negative regulation of activation in cells of the immune system that expresses TIM-3 is achieved by inhibiting the NF-Kb transcription factor, thus blocking the TLR-mediated response (49). Research has also documented a reduction in the release of pro-inflammatory cytokines, such as IFN-γ and IL-12, in macrophages with higher TIM-3 expression, compared to macrophages with a low expression of this molecule (33, 35). Finally, it has been demonstrated that the blocking or silencing of TIM-3 favors the development of sepsis, a severe inflammatory condition with a high mortality rate (49).

This evidence leads us to suggest that the TIM-3 molecule may play a dual role, since it participates in the activation of infected macrophages by inducing IL-1b-dependent bacteriostatic and bactericidal mechanisms. Also, when expressed in T lymphocytes, TIM-3 can negatively regulate the Th1 immune response. It is not yet possible to clarify the factors that produce these apparently contradictory findings and the possibility exists that the mechanisms induced by TIM-3 could occur simultaneously. The level of TIM-3 expression may be an important factor that affects the results and function of the TIM-3-dependent pathway. No simultaneous or comparative studies have yet been conducted to analyze the role of TIM-3 in macrophages and other cells of the innate immune system in the context of bacterial infections.

## Conclusion

Since the discovery of the TIM-3 molecule, numerous studies performed have made it possible to determine some aspects of the functioning of this molecule. At present, TIM-3 is considered an inhibitory molecule of the activation of diverse cells, or an immune repressor system. On the other hand, there is evidence that TIM-3 can favor the activation of some mechanisms of the immune response, as reported for macrophages. Although these findings seem contradictory, one could propose that the level of TIM-3 expression is an important factor that must be considered, since studies have shown that a reduction in the level of expression of TIM-3 favors the production of IL-12 and the activation of monocytes and macrophages (33). Although research has shown that the union of Gal-9 with TIM-3 induces activation of caspase-1 and the posterior release of IL-1b, we do not yet know whether this mechanism is reproduced in the diverse sub-populations of macrophages, or even monocytes, since TIM-3 expression is dynamic and there are antecedents indicating that high expression of this molecule produces a state of tolerance or anergy to diverse antigenic stimuli. We require studies designed to evaluate the mechanisms of activation of phagocytic cells that take into account the level of expression and signaling of TIM-3. Finally, no one has yet demonstrated that TIM-3 is associated with the Bat-3 (14) protein in macrophages and DCs nor its interaction with the Lck protein, a very important kinase for the activation of transcription factors such as NF-kb. Analyzing and clarifying these issues will provide a broader perspective on the functions of TIM-3 and its role in myeloid cells. Such research is important because this field of study is lagging slightly behind analyses of the TIM-3 molecule in lymphoid cells.

## Author Contributions

AO-G: text and drawings. IS-O: design and text. LT-B and IS-O: text revision and final approval.

## Conflict of Interest Statement

The authors declare that the research was conducted in the absence of any commercial or financial relationships that could be construed as a potential conflict of interest.

## Abbreviations

ARG-1, arginase 1; BAT-3, HLA-B 3-associated transcript; CD, cluster of differentiation; cDNA, complementary deoxyribonucleic acid; DC, dendritic cell; DNA, deoxyribonucleic acid; EAE, experimental autoimmune encephalitis; Gal-9, galectin 9; HMGB1, high mobility group box 1 protein; huHAVcr-1, human homolog of the receptor of the hepatitis A 1 virus; IFN-γ, gamma interferon; IgV, immunoglobulin domain; IL, interleukin; iNOS, inducible nitric oxide synthase; ITAM, tyrosine-based activation motif; LPS, lipopolysaccharide; M.tb, *Mycobacterium tuberculosis*; M-CSF, macrophage colony-stimulating factor; MMP-9, matrix metalloproteinase 9; NK, natural killer cell; PD-1, programed death receptor 1; RNA, ribonucleic acid; siRNA, interference ribonucleic acid; STAT, signal transducer and transcription activator; TAM, tumor-associated macrophage; TB, tuberculosis; Tc, cytotoxic T lymphocyte; TGF-β, transforming growth factor beta; Th, T lymphocyte cooperator or collaborator; TIM-3, T cell molecule with immunoglobulin and mucin domain 3; TLR, toll-like receptor; TNF-α, tumor necrosis factor alpha; VEGF, vascular endothelial growth factor; VIH, human immunodeficiency virus.
